# Molecular advances in the cell biology of SARS-CoV and current disease prevention strategies

**DOI:** 10.1186/1743-422X-2-35

**Published:** 2005-04-15

**Authors:** Caren J Stark, CD Atreya

**Affiliations:** 1 Division of Viral Products, Center for Biologics Evaluation and Research, US Food and Drug Administration, Bethesda, MD 20892 USA

**Keywords:** Antivirals, Cell biology, Molecular virology, SARS-CoV, Vaccines

## Abstract

In the aftermath of the SARS epidemic, there has been significant progress in understanding the molecular and cell biology of SARS-CoV. Some of the milestones are the availability of viral genome sequence, identification of the viral receptor, development of an infectious cDNA clone, and the identification of viral antigens that elicit neutralizing antibodies. However, there is still a large gap in our understanding of how SARS-CoV interacts with the host cell and the rapidly changing viral genome adds another variable to this equation. Now the SARS-CoV story has entered a new phase, a search for preventive strategies and a cure for the disease. This review highlights the progress made in identifying molecular aspects of SARS-CoV biology that is relevant in developing disease prevention strategies. Authors conclude that development of successful SARS-CoV vaccines and antivirals depends on the progress we make in these areas in the immediate future.

## Introduction

Following reports of the last case of the **s**evere **a**cute **r**espiratory **s**yndrome (SARS) epidemic in July 2003, there has been remarkable progress in several areas of research on the molecular identification of the pathogen and its pathogenesis, replication, genetics, and host immunogenicity, as well as elegant epidemiological studies. The sequence of epidemiological events that unfolded early in the outbreak gave researchers a glimpse into the first new pathogen of the era of globalization. As the year 2002 drew to a close, multiple reports of an "infectious atypical pneumonia" caught public health officials across the globe by surprise and suggested that a new human pathogen had emerged in the Guangdong Province in China [1]. By the end of February 2003, this outbreak of SARS had infected almost 800 patients and caused 31 deaths in the Province [2]. One month later, the disease had spread throughout Asia and into Europe and North America. This epidemic eventually affected more than 8000 people and resulted in approximately 800 deaths worldwide, with mortality rates reaching over 40% in certain populations [3,4].

Electron microscope analysis quickly identified the putative SARS agent as having features associated with coronaviruses. The SARS agent was later unambiguously identified as a new coronavirus member and named SARS-coronavirus (SARS-CoV) [5-7]. Coronaviruses are enveloped, plus-stranded RNA viruses with the largest RNA genomes known (on the order of 30 kb). Coronaviruses have long been important in the world of veterinary viral diseases. However, previously known human coronaviruses such as HCoV-229E and HCoV-OC43 cause only minor health problems such as the common cold and gastrointestinal diseases. In contrast, the SARS-CoV pathogen causes fever, pulmonary edema, and diffuse alveolar damage in severely affected individuals (collectively termed severe acute respiratory syndrome) [8]. SARS-CoV is also a unique coronavirus in that, to date, it is the only member known to cause severe morbidity and mortality in humans [8]. Demonstration that SARS-CoV can cause serious public health problems has focused attention on the need to understand the viral replicative strategy and devise prophylactic measures.

The clinical symptoms of SARS are those of a lower respiratory tract infection and are accompanied by damage to the lungs [6,9,10]. Gastrointestinal involvement is also common, with more than 20% of patients presenting with watery diarrhea [11]. Fecal samples from SARS patients taken up to 25 days after onset of disease contain viral RNA, which suggests viral shedding through the bowels [5]. Liver dysfunction has also been reported based on observed necrosis in hepatocytes [9,12]. Post-mortem tissue examination of SARS patients has found the virus presence in lung, bowel, lymph node, liver, heart, kidney, and skeletal muscle samples [13]. The primary mode of SARS-CoV transmission is airborne via droplets [14,15]. However, there are also reports of the presence of replicating virus in blood cells (peripheral blood mononuclear cells) and in the small and large intestine [11,16]. Alternative modes of transmission, such as blood-borne or fecal-oral are therefore possible.

The virus has been isolated from wild animals (Himalayan palm civets and raccoon dogs) found in the animal markets of Guangdong, China [17]. The actual natural reservoir for SARS-CoV is still unknown. Once transmitted to humans, SARS-CoV appears to evolve to facilitate to human-human transmission. Sequence analysis of different SARS-CoV isolates from early in the epidemic show deletion events occurring in open reading frame 8 (Orf 8) [18]. Identical deletions in Orf 8 have also been seen in animal coronaviruses supporting the idea that SARS-CoV was introduced to humans via an animal intermediate. In addition to deletion events occurring early and late in the epidemic, a slowing of missense mutations is seen over time, with the most extensive changes occurring in the S protein during the early stages of the outbreak [18]. This suggests the virus has undergone some level of adaptation but has ultimately stabilized at a time in the epidemic where SARS-CoV has become more virulent. Deciphering the evolutionary passage of this virus will undoubtedly provide valuable information on preventing future outbreaks.

In the wake of the SARS epidemic, a number of excellent review articles on the clinical and molecular aspects of SARS epidemiology have been published. These reviews have focused primarily on rapid advances made in the identification and characterization of SARS-CoV genomes as well as describing the etiology of the virus and clinical features of the disease [19-21]. Now the SARS-CoV story has entered a new phase, a search for preventative strategies and a cure. In this review, we highlight the progress made in revealing the molecular aspects of SARS-CoV biology and how such information may lead to strategies for disease prevention.

## Brief overview of the SARS-CoV genome

Coronaviruses are subdivided into three groups based on genetic and serological markers [22]. Groups I, and II infect mammals while group III is specific for avian species. Group I members are the porcine transmissible gastroenteritis virus (TGEV) and epidemic diarrhea virus (PEDV), feline and canine coronavirus (FCoV and CCoV), and human coronavirus 229E (HCoV-229E). Group II includes porcine hemagglutinating encephalomyelitis virus (HEV), murine hepatitis virus (MHV), bovine, equine, and rat coronavirus (BCoV, ECoV, and RtCoV), and human coronavirus OC43 (HCoV-OC43). Group III includes the turkey coronavirus (TCoV), pheasant coronavirus and avian infectious bronchitis virus (IBV). Although most closely related to Group II coronaviruses, SARS-CoV, with some of its unique genetic features, represents a distinct phylogenetic group [22-24].

To date, approximately 61 SARS-CoV genomic sequences have been analyzed representing different phases of the epidemic (early, middle, and late) and two isolates obtained from palm civets [18]. The SARS-CoV genomic RNA is approximately 30 kb and is organized into 13 to 15 open reading frames (ORFs) [25-27]. The SARS CoV structural gene arrangement follows the same pattern as most coronavirus genomes: 5'- Replicase (ORF 1a)-Protease (ORF 1b)-Spike (S)-Envelope (E)-membrane (M)-Nucleocapsid (N)-3' [27]. However, in contrast to other coronaviruses, two ORFs of unknown function are located between the S and E ORFs and 3–5 ORFs are located between M and N. In addition, despite the evolutionary overlap between SARS-CoV and Group II coronavirus genome sequences, the SARS genome lacks a gene for hemagglutinin-esterase (HE) protein, which is common to a majority of Group II coronaviruses [25]. For an excellent pictorial representation of SARS-CoV genome with functions (or lack of) assigned to each ORF, please refer to the recent review by Tan et al [21]. A significant milestone in SARS-CoV molecular biology was the construction of a SARS-CoV full-length cDNA-containing plasmid from which infectious viral RNA can be produced [28]. This development facilitates the study of SARS-CoV gene functions and should promote the elucidation of function for ORFs whose function is still unknown [29]. Although it has been the perception that these ORFs are not essential for viral replication, they may play a role in the manifestation or severity of disease.

## Progress in SARS-CoV genome-based evolutionary biology

RNA viruses utilize a variety of mechanisms to exchange their genetic repertoire. The viral RNA dependent RNA polymerases (RdRP) have a built in error rate that allows diversification of the genomic sequence as replication proceeds. Estimates put the error rate of an RdRp at 10^-3 ^to 10^-5 ^per nucleotide [30]. Coronaviruses also undergo high rates of RNA recombination, providing an additional mechanism by which the viruses can rapidly amplify genomic diversity. The SARS-CoV polymerase gene has a recombination breakpoint, suggesting multiple genetic origins for this molecule. [31]. These evolutionary mechanisms may have facilitated the adaptation of the animal-borne SARS-CoV ancestor to the human host, suggesting that such events in the future could lead to a virus with increased pathogenicity for humans or one capable of infecting multiple species. Recent evidence indicates that the human-adapted SARS virus has crossed into another species. Sequence and epidemiological analyses revealed that a SARS-CoV isolated from a pig was derived from a human strain. Complete nucleotide sequencing of the pig virus isolate (designated TJF) and an S gene-based phylogenetic tree analysis revealed a closer relationship with human SARS-CoV isolates than with animal coronaviruses [32].

## Progress in cell biology of SARS-CoV: Signaling pathways

Successful viral replication depends upon the ability of the virus to subvert cellular processes to their advantage and counteract cellular defense mechanisms. Such virus-cell interactions represent potential targets for the development of virus-specific antiviral drugs, therapeutics, and prophylactic vaccines. Different viruses, based on their target cell types and entry pathways, differ in their cellular exploitation mechanisms. The mechanism of SARS virus pathogenesis *in vivo *may reflect both the effect of viral replication in target cells and host immune responses. The molecular basis for SARS-CoV replication, the signaling pathways affected, and the inflammatory responses provoked by viral infection are not yet clearly understood. Progress in these areas should lead to more effective preventive strategies to counter SARS-CoV infections.

**Figure 1:**
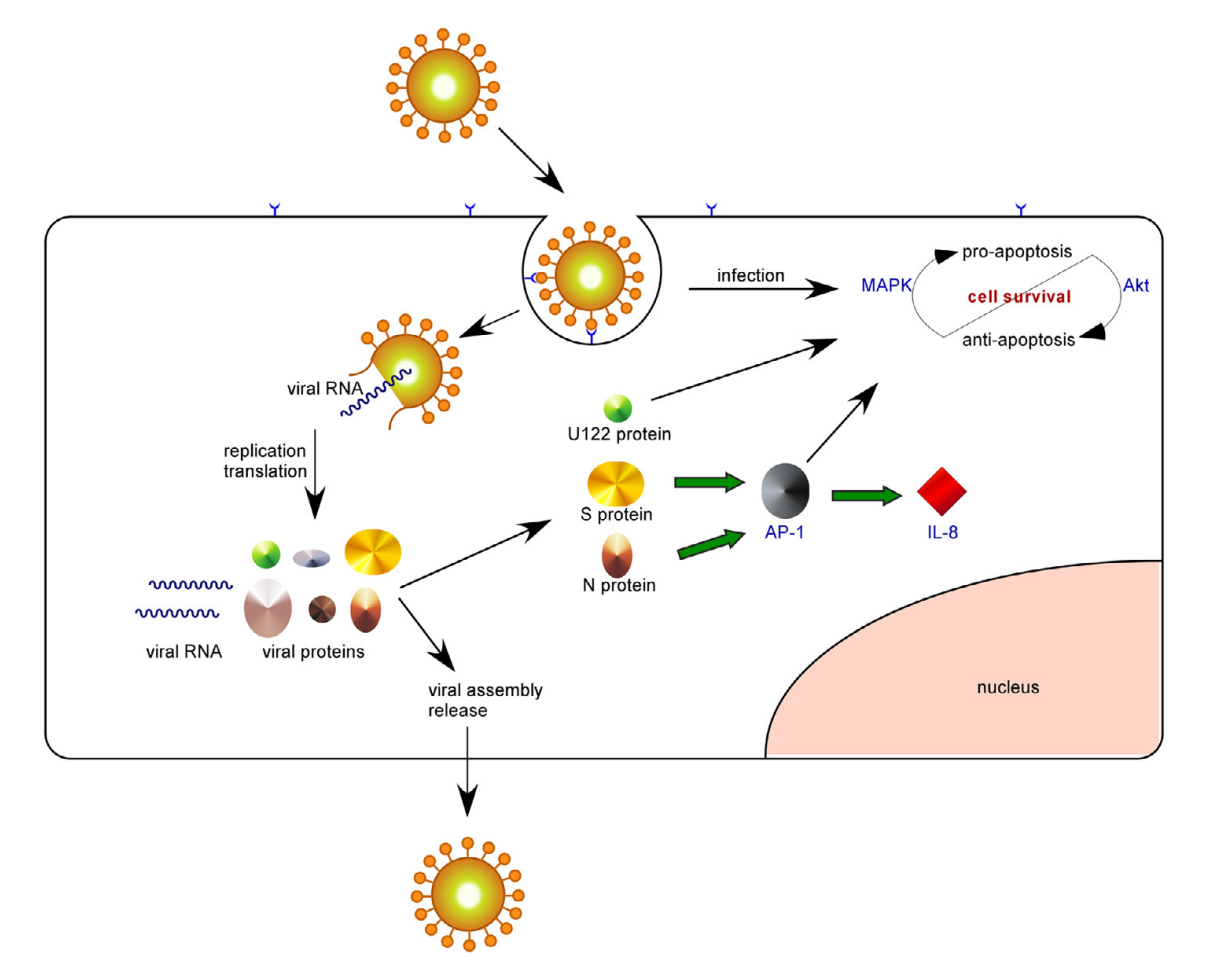
The balance of cell survival and cell death in response to SARS-CoV infection. SARS-CoV is shown approaching a cell with ACE2 receptors (blue "Y"s) on the surface. The virus enters the cell, uncoats, and the viral RNA is replicated and translated. The SARS-CoV U122 protein induces apoptosis in cells. SARS-CoV S and N proteins each can activate the cellular AP-1 protein, which regulates apoptosis, as well as other cellular processes. AP-1 also activates IL-8, a cellular cytokine. SARS-CoV infection induces both MAPK (pro-apoptotic) and Akt (anti-apoptotic) pathways. How this balance between cell survival and apoptosis is maintained is yet unknown. Cellular proteins are labeled in blue, viral proteins in black.

It has been shown that the SARS-CoV N protein selectively activates the Activator Protein-1 (AP-1) signal transduction pathway, which regulates a wide variety of cellular processes including cell proliferation, differentiation, and apoptosis [33]. Such viral induced modifications of the AP-1 pathway may play a significant role in the viral replicative strategy. Recently, another group demonstrated that the S protein alone induces AP-1 activation and that the region from 324–688 amino acids within the S protein is essential for AP-1 activation-dependent IL-8 induction [34]. Another SARS-CoV protein, the U122 ORF of unknown function (also known as X4), was shown to be produced in virus infected Vero E6 cells and expression of this protein alone was shown to induce apoptosis in cell culture [35,36]. This raises the question of how apoptosis of SARS-CoV infected cells is balanced in order for the virus to survive and propagate (Figure 1). This has been addressed to some extent in recent studies which indicate that SARS-CoV infection of Vero E6 cells induces both pro-apoptotic [activation of p38 mitogen-activated protein kinase (MAPK)] and anti-apoptotic [activation of the protein kinase B (PKB, also known as Akt)] signaling pathways, although Akt induction appears to be insufficient to prevent the virus-induced apoptosis [37,38]. Exactly how SARS-CoV manipulates these cellular signaling pathways to facilitate viral replication remains to be determined.

As mentioned above, IL-8 induction was shown to be dependent upon AP-1 activation by SARS-CoV S protein and in this process NF-κB was not involved [34]. This may partially explain the clinical observation of dramatic cytokine storm (high serum levels of IL-6 and IL-8) and inflammation responses observed in SARS patients in the acute stage associated with lung lesions; it has been also suggested that the elevations of IL-6 and IL-8 due to SARS-CoV infection of the respiratory tract can induce the hyper-innate inflammatory response [39]. It is established that cellular MAPKs regulate AP-1 activation-dependent IL-8 induction in viral infections [40-42]. In SARS-CoV infection, the IL-8 induction signaling pathway is perhaps related to angiotensin-converting enzyme 2 (ACE2), as anti-ACE2 antibodies inhibit IL-8 induction/release [34]. ACE2 is the cellular receptor for the SARS-CoV and the receptor-binding sites on the virion are located in the 12–672 amino acid region of the S protein [43].

## Current advances towards SARS-CoV prevention strategies

During the SARS outbreak that occurred in 2002–2003, the spread of the disease was primarily controlled by strict quarantine protocols and patient-isolation measures as well as by broad-spectrum antibiotics and antiviral regimens with or without administration of corticosteroids [44,45]. Since then, the wealth of information that has emerged on SARS-CoV molecular and cellular biology, as updated in the preceding sections of this review, now offers potential avenues for developing more efficient anti-viral as well as vaccine strategies.

### a. Antiviral agents

Coronavirus genome structure and major gene-product functions have been known for years, but since they cause mild disease, selection of the virus-specific antiviral drugs was not a priority in the past. The SARS-CoV epidemic changed this selective view. Tan et al, 2004, tabulated a screen of available antiviral agents against SARS virus in detail in their recent review [46]. The obvious molecular targets for SARS-CoV antiviral agents are the viral polymerase/replicase, protease, receptor, the viral mRNA cap-1 methyl transferase and NTPase/helicase [47-54]. In addition, a 32-nucleotide long, highly conserved RNA structure in the 3' untranslated region of coronaviruses and astroviruses was identified [55]. This structure resembles the 530 loop of 16s rRNA involved in translation initiation suggesting a possible role for this element in sequestering host translation machinery. The tertiary interactions of this structure create a tunnel lined with negative charge where Mg^2+ ^can bind. This unique structure presents an attractive target for tunnel binding antiviral drugs [55]. Finally, since the functional details of most coronavirus replicase gene products are not known, random screening of potential antiviral compound libraries will be a key area of drug discovery for SARS virus in the near future [47].

### b. Vaccine development

Vaccines are the best and least expensive prophylactic measures against pathogens that cause epidemics in humans. The fact that high titers of virus neutralizing antibody to SARS-CoV are found in sera of patients recovering from infection and that those infected with the virus show improvement after passive antibody administration suggests a SARS-CoV vaccine is possible and points toward antibody based treatments for the disease [47,56-58]. However, in developing SARS CoV vaccines, there are lessons to be learned from the world of veterinary CoV vaccines. In a review by Saif, it was pointed out that coronaviruses in general target mucosal surfaces and therefore eliciting local (mucosal) immunity is a major consideration in the development of SARS-CoV vaccines; this largely depends on the type of vaccine, delivery systems, and immuno-modulatory adjuvants used [59]. Further, immunity against animal CoV is usually short term, necessitating periodic boosting, which in the end may not be sufficient to prevent re-infection.

Despite these potential pitfalls in the development of a human vaccine, efforts to develop a vaccine to prevent another SARS outbreak are underway. Several laboratories around the globe are working at an unprecedented pace to develop a SARS vaccine utilizing essentially two different types of SARS-CoV-derived immunogens, 1) inactivated whole virus, and 2) SARS-CoV encoded N and S proteins using recombinant DNA methods. The possibility of producing an engineered live, attenuated SARS-CoV has also been considered.

#### 1. Inactivated whole virus

Takasuka et al (2004) have reported that subcutaneous administration of UV-inactivated purified SARS-CoV virion elicits a high level of humoral immunity, resulting in long-term antibody secretion and memory B cells [60]. The antibodies elicited in mice recognized both the spike (S) and nucleocapsid (N) proteins of the virus. The inactivated virus also induced regional lymph node T-cell proliferation and significant levels of cytokine production upon restimulation with inactivated virus in vitro [60]. These studies suggest that whole-killed virion may have the potential as a candidate antigen for SARS vaccine to elicit both humoral and cellular immunity. When SARS-CoV inactivated by beta-propiolactone was used as antigen in mice and rabbits, the animals elicited antibodies against the receptor-binding domain (RBD) present in the S1 region of SARS-CoV. These antibodies effectively inhibited the S-protein mediated SARS-pseudovirus entry up to 50%, suggesting the potential of the inactivated SARS-CoV as antigen for vaccine development [61]. Depletion of RBD-specific antibodies from patient or rabbit immune sera by immunoadsorption, significantly reduced the virus neutralizing ability of the sera, suggesting that the RBD epitope in the S protein is a critical determinant in developing vaccine strategies [62].

#### 2.1. Cloned N protein

The N protein of SARS-CoV appears to be more conserved than S and M proteins and it has been suggested that this protein may play a role in cell-mediated immunity in SARS-CoV infections and also is an important viral antigen for the early diagnosis. Vaccination of C57BL/6 mice with a SARS-CoV N protein expressed by an E1/partially E3-deleted, replication-defective human adenovirus 5 vector was shown to produce potent SARS-CoV-specific humoral and T cell-mediated immune responses, suggesting the potential of this construct to be used as SARS-CoV vaccine [63]. Along the same line, intra-muscular immunization of BALB/c mice with a plasmid DNA construct encoding the full-length N protein was shown to elicit serum anti-N antibodies and spenocyte proliferative responses against the N protein [64]. The immunized mice also produced strong delayed-type hypersensitivity (DTH) and CD8 (+) CTL responses to the N protein, suggesting that the N protein is not only an important B cell immunogen, but also can elicit broad-based cellular immune responses [64]. In another novel strategy, the N protein was expressed in the cytoplasm of *Lactococcus lactis *bacterium and the N-expressing bacteria were administered to mice by intranasal or oral route [65]. In this case, significant levels of N-specific IgG in the mice sera were detected, suggesting that the engineered bacteria may serve as a mucosal vaccine against SARS-CoV [65].

#### 2.2. Cloned viral S spike protein or, S-containing pseudovirions

Although immunization with inactivated viral vaccine provides significant protection in animals against challenge with certain corresponding pathogenic CoVs, in the case of SARS-CoV there remains the threat of introducing live virus into the environment from partially inactivated vaccine, as there are no validated and effective inactivation measures developed yet. To circumvent this obstacle, Chen et al have introduced the S protein into the deletion III region of the live, attenuated modified vaccinia virus Ankara (MVA) vector [66]. This recombinant virus elicits potent neutralizing antibodies in mice, rabbits, and monkeys and the major epitope is mapped to the virus receptor-binding region [66]. In another approach, it has been demonstrated that co-expression of SARS-CoV S, M and N expression plasmids in human 293T cells result in the formation of SARS-CoV pseudoparticles (virus-like particles or VLPs) [67]. These findings help us understand the viral morphogenesis as well as offer a safer alternative to using live, replicating SARS virus in the development of vaccines.

#### 3. Attenuated live virus

The third possibility is a genetically engineered version of live SARS-CoV for traits such as attenuated phenotype, increased immunogenicity, and safe handling (out of BL3+ facility). A full-length SARS-CoV cDNA-containing plasmid has been developed from which synthetic infectious viral RNA can be produced [28]. This system allows for the functional analysis of each gene in the context of infection and can be used for making attenuated strains for vaccine development.

## Conclusions: Limitations to current SARS vaccine strategies

SARS-CoV clearly has pandemic potential. Although progress in SARS-CoV molecular and cell biology research has been remarkable, there remain clear limitations regarding vaccine development due to a lack of complete understanding in the areas of animal models of the disease as well as host immune responses to the evolving molecular diversity of this newly emerged human virus. Caution is warranted when utilizing experimental data originating from one SARS-CoV strain infection in one animal species or cell line in the development of a human vaccine. The rapid development of an effective SARS-CoV vaccine depends upon continuing basic research.

A study on the evolving S protein molecular diversity in SARS-CoV isolates and its unexpected profound immuno-functional effects illustrates this point [68]. The S protein exhibited minor genetic diversity among 8 strains transmitted during human outbreaks in early 2003. Synthetic versions of these S variants with human preferred codons were tested for 1) their ability to bind the receptor (hACE-2), and 2) their sensitivity to antibody neutralization with viral pseudotypes. In these sets of experiments, substantial functional differences were found in S derived from a Guangdong province case -isolate and two palm civets isolates. Antibodies that neutralized most human isolates-derived S proteins unexpectedly enhanced entry mediated by the civet virus-derived S proteins [68]. This novel observation emphasizes the need to understand the molecular potential of the SARS-CoV genome in developing vaccines to prevent human disease. As mentioned previously, studies also point to the fact that variability in the S protein from early to late disease outbreak stages has been detected [18]. There is a large gap in our understanding of how SARS-CoV interacts with the host cell and the rapidly changing genome of SARS-CoV indicates the potential variability of such interactions [25]. Development of successful vaccines against SARS virus therefore depends on the progress we make in these areas in the immediate future.

## Competing Interests

The author(s) declare that they have no competing interests.

## Authors' Contributions

Authors contributed equally to the intellectual content of this review article.

## Disclaimer

The views presented in this article do not necessarily reflect those of the Food and Drug Administration or United States government.
